# Supplementary figures and images for: Assessing BRCA1 activity in DNA damage repair using human induced pluripotent stem cells as an approach to assist classification of BRCA1 variants of uncertain significance
Source: PLoS One. 2021 Dec 2;16(12):e0260852. doi: 10.1371/journal.pone.0260852 (PMC8638976; doi:10.1371/journal.pone.0260852)

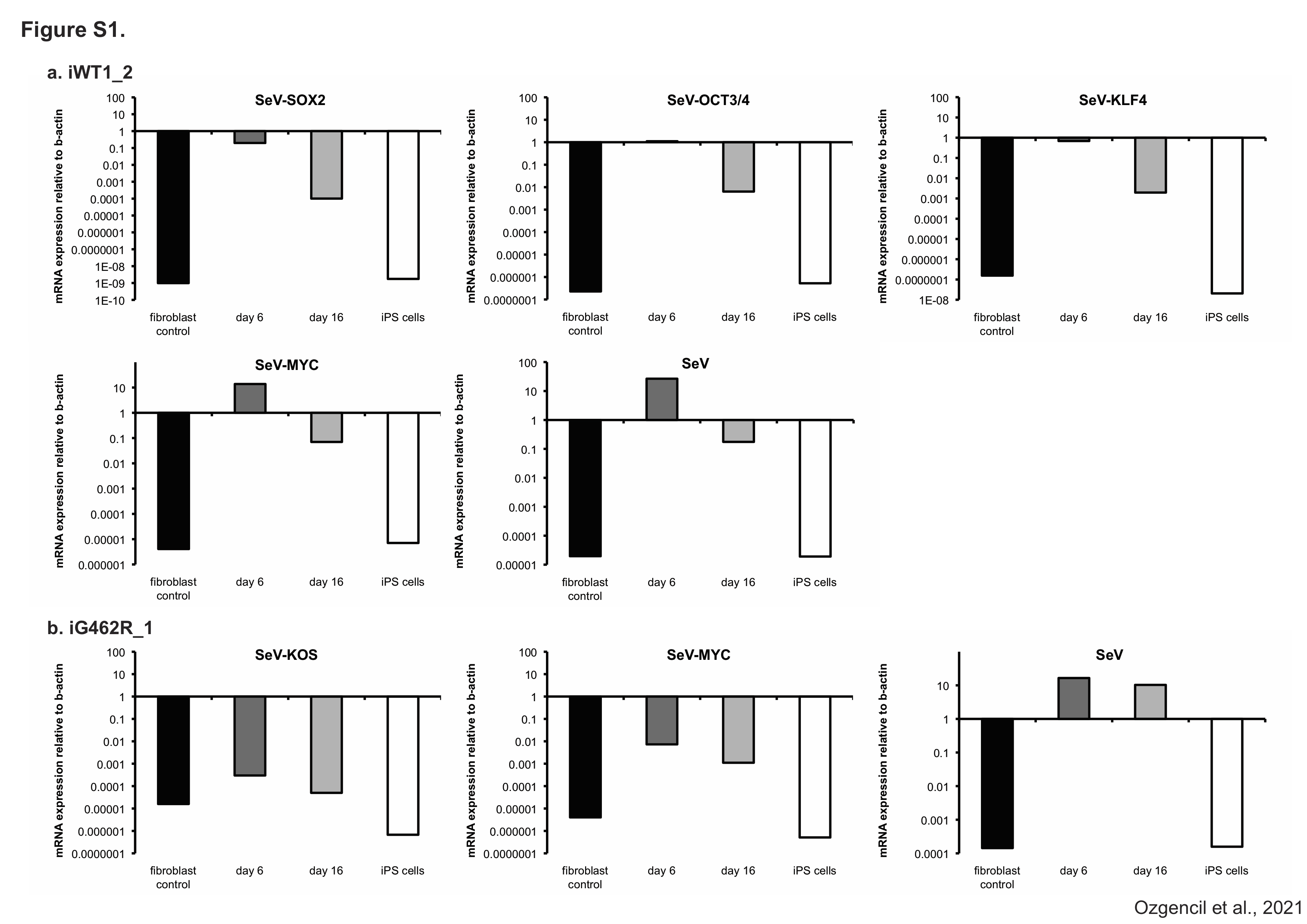

Supplement: S1 Fig — Taqman qPCR analysis of reprogramming factor and SeV sequences in a iWT1_2. b iG462R_1. c iC61G_2. d iD1733G_1. e iK381X_1. f iV1687G_1. g iY856H_2. h iQ1811K_1. i iA1708E_1 iPS cells compared to uninfected fibroblasts. (ZIP) [file pone.0260852.s001.zip › S1ab_Fig.tiff]

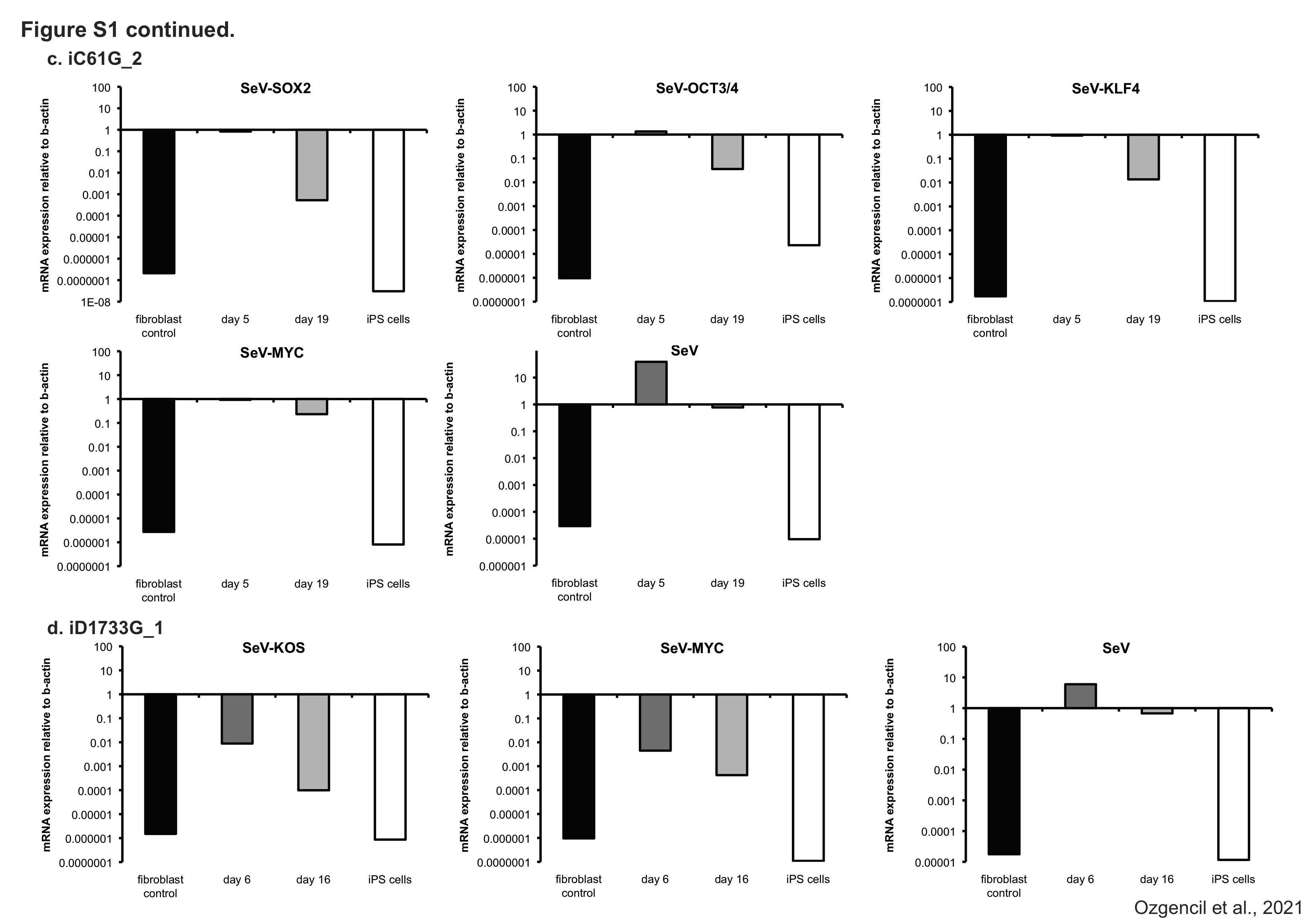

Supplement: S1 Fig — Taqman qPCR analysis of reprogramming factor and SeV sequences in a iWT1_2. b iG462R_1. c iC61G_2. d iD1733G_1. e iK381X_1. f iV1687G_1. g iY856H_2. h iQ1811K_1. i iA1708E_1 iPS cells compared to uninfected fibroblasts. (ZIP) [file pone.0260852.s001.zip › S1cd_Fig.tiff]

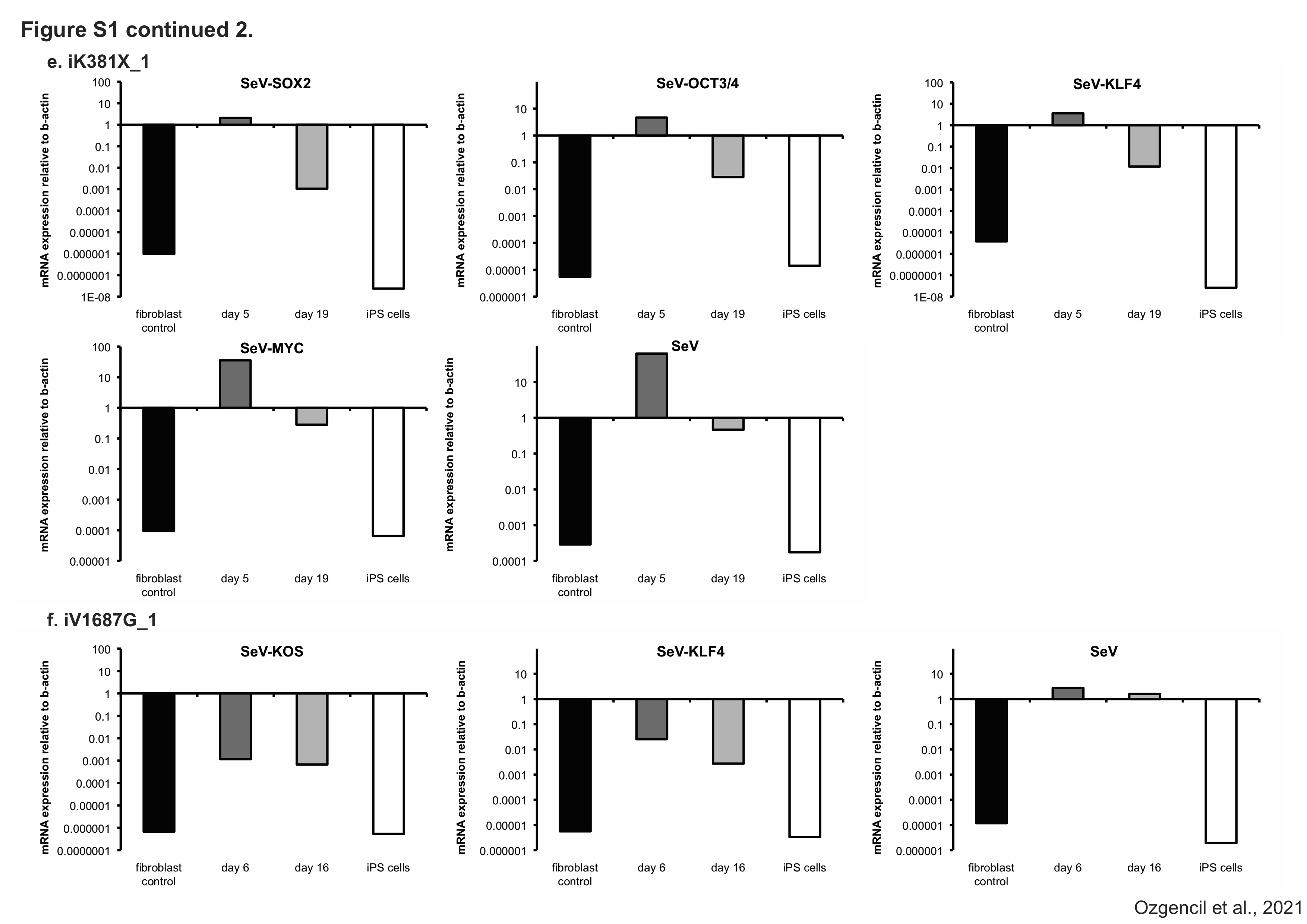

Supplement: S1 Fig — Taqman qPCR analysis of reprogramming factor and SeV sequences in a iWT1_2. b iG462R_1. c iC61G_2. d iD1733G_1. e iK381X_1. f iV1687G_1. g iY856H_2. h iQ1811K_1. i iA1708E_1 iPS cells compared to uninfected fibroblasts. (ZIP) [file pone.0260852.s001.zip › S1ef_Fig.tiff]

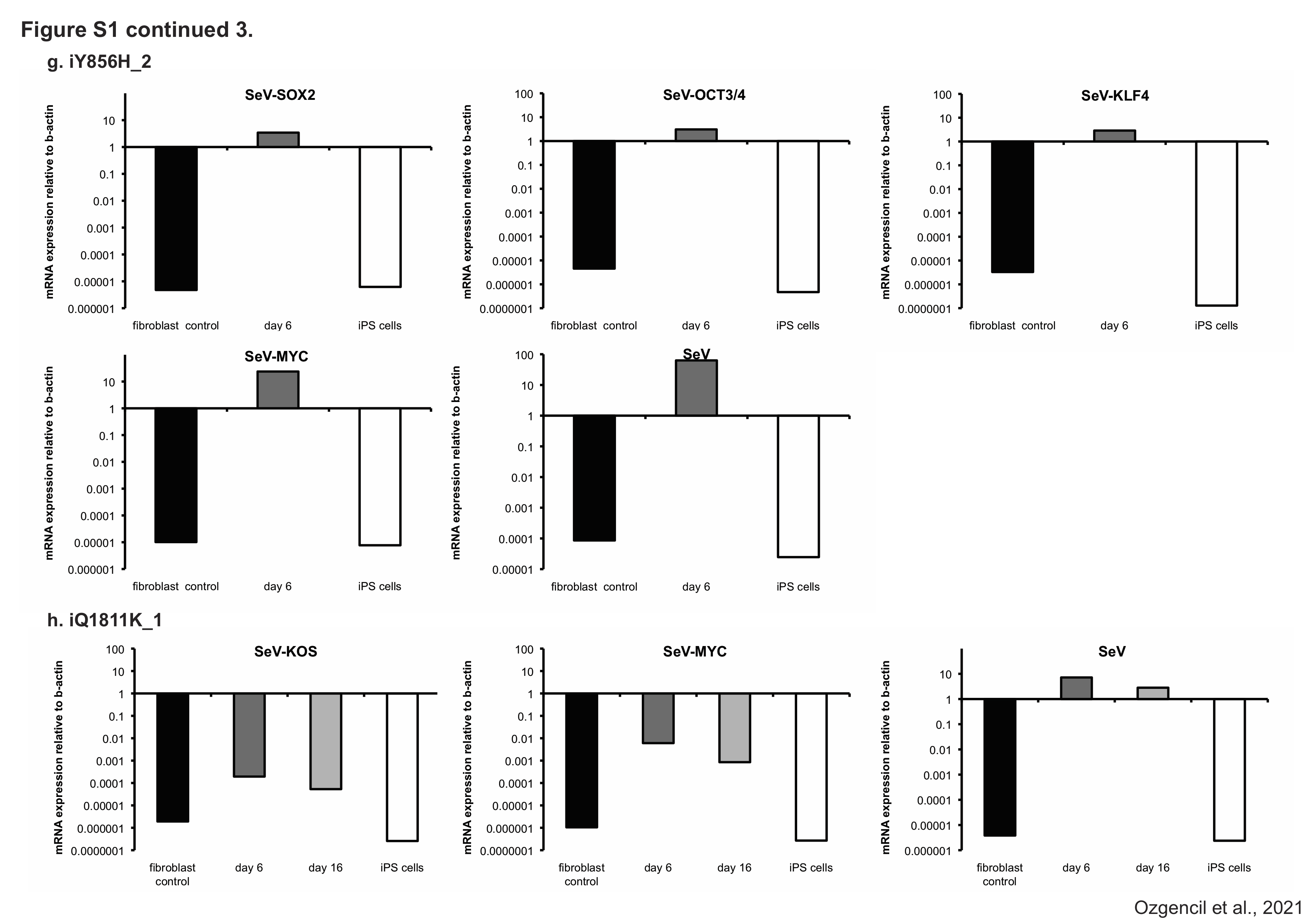

Supplement: S1 Fig — Taqman qPCR analysis of reprogramming factor and SeV sequences in a iWT1_2. b iG462R_1. c iC61G_2. d iD1733G_1. e iK381X_1. f iV1687G_1. g iY856H_2. h iQ1811K_1. i iA1708E_1 iPS cells compared to uninfected fibroblasts. (ZIP) [file pone.0260852.s001.zip › S1gh_Fig.tiff]

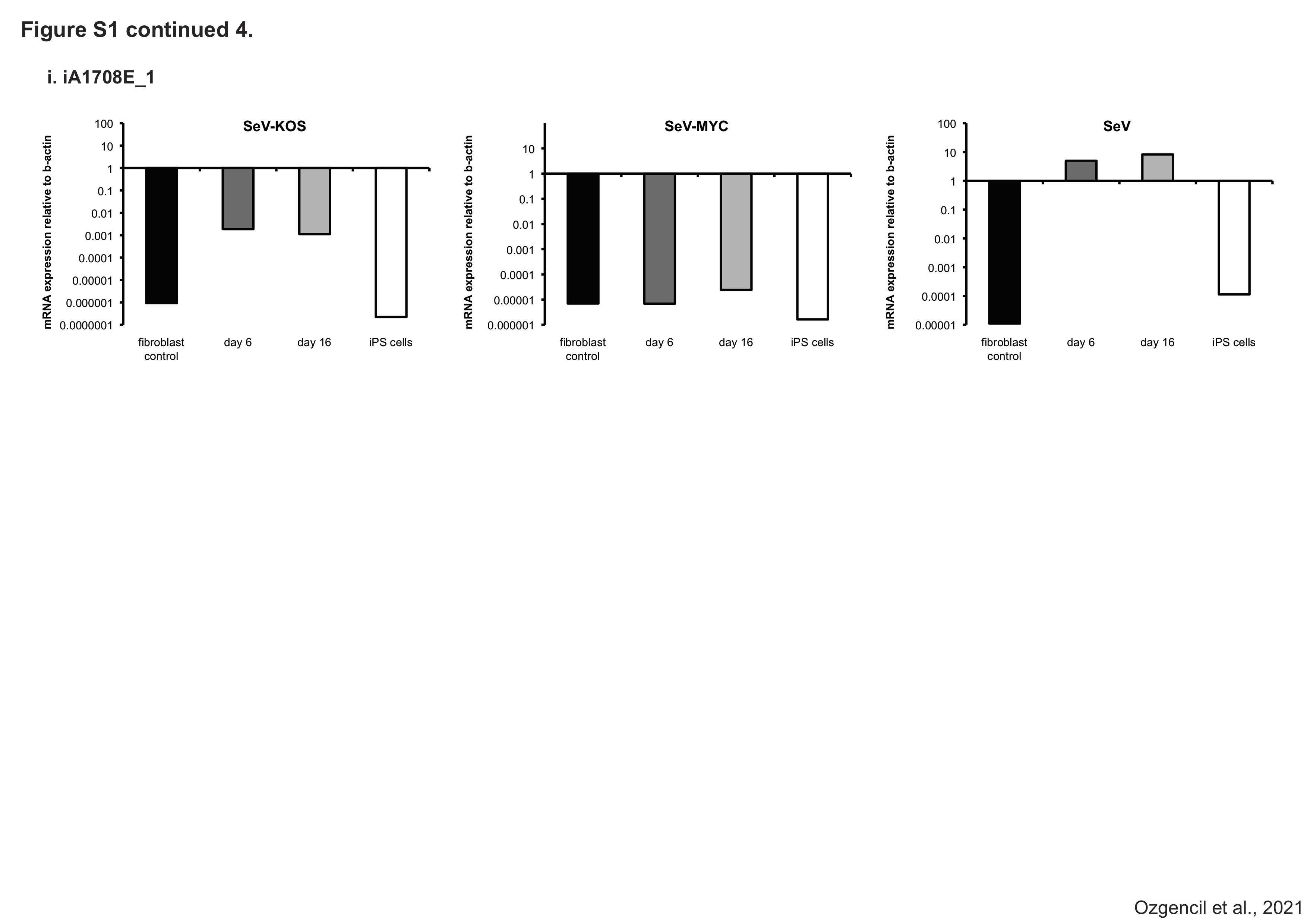

Supplement: S1 Fig — Taqman qPCR analysis of reprogramming factor and SeV sequences in a iWT1_2. b iG462R_1. c iC61G_2. d iD1733G_1. e iK381X_1. f iV1687G_1. g iY856H_2. h iQ1811K_1. i iA1708E_1 iPS cells compared to uninfected fibroblasts. (ZIP) [file pone.0260852.s001.zip › S1i_Fig.tiff]

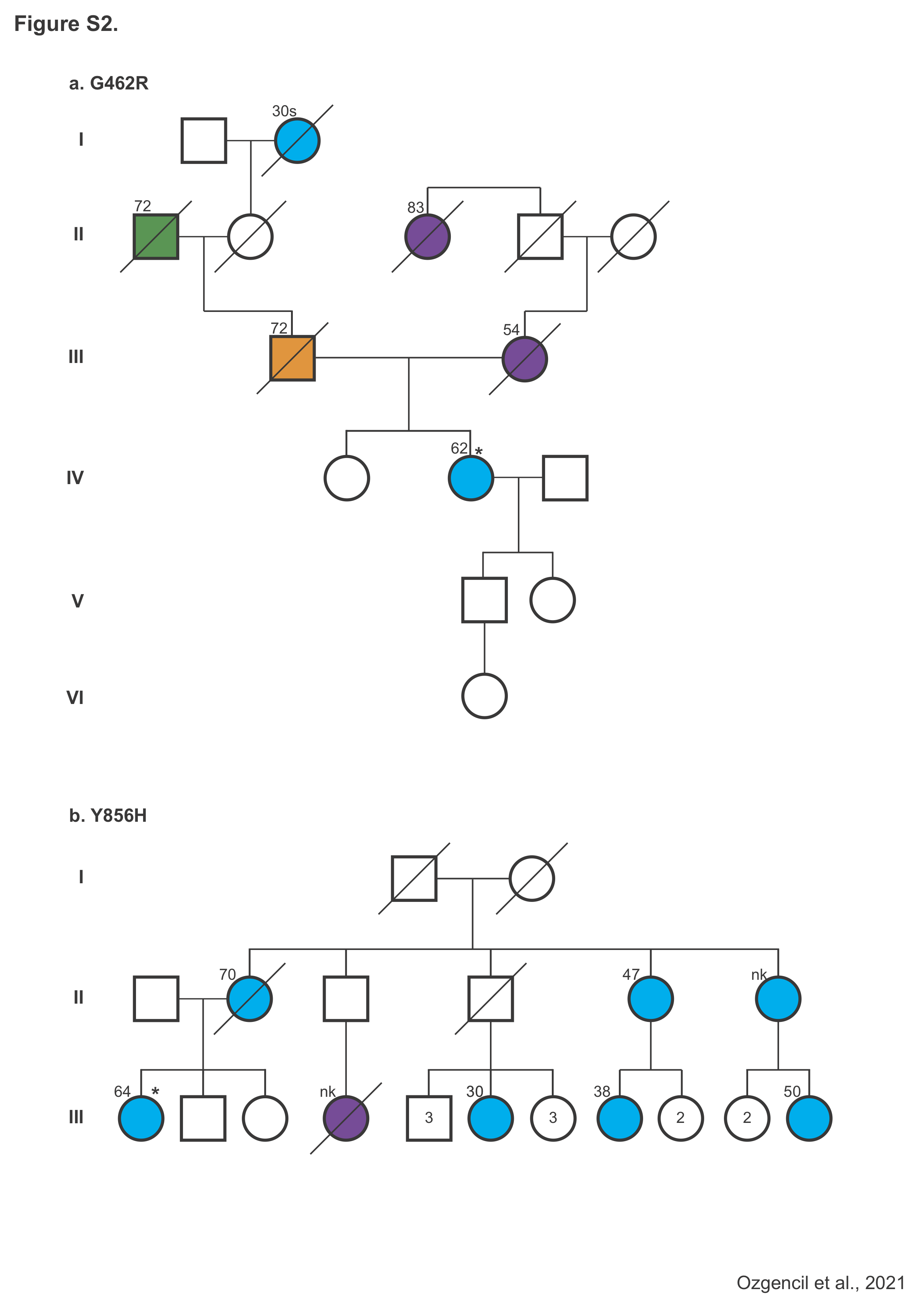

Supplement: S2 Fig — a The six-generation pedigree of the BRCA1 G462R family. b The three-generation pedigree of the BRCA1 Y856H family. c The four-generation pedigree of the BRCA1 D1733G family. d The four-generation pedigree of the BRCA1 Q1811K family. e The five-generation pedigree of the BRCA1 V1687G family. Patient ages at the time of cancer occurrence are located to the upper left of each symbol. Symbols coloured blue or purple indicate patients with breast cancer or ovarian cancer, respectively; pink indicates skin cancer, green indicates lung cancer, orange indicates prostate cancer, dark red indicates leukaemia, and grey denotes cancers with an unknown primary site. A diagonal indicates deceased individuals. Numbers inside symbols indicate multiple individuals. Asterisks (*) identify patients whose biopsies were used for iPS cell derivation. Nk = age at the time of cancer not known. (ZIP) [file pone.0260852.s002.zip › S2ab_Fig.tiff]

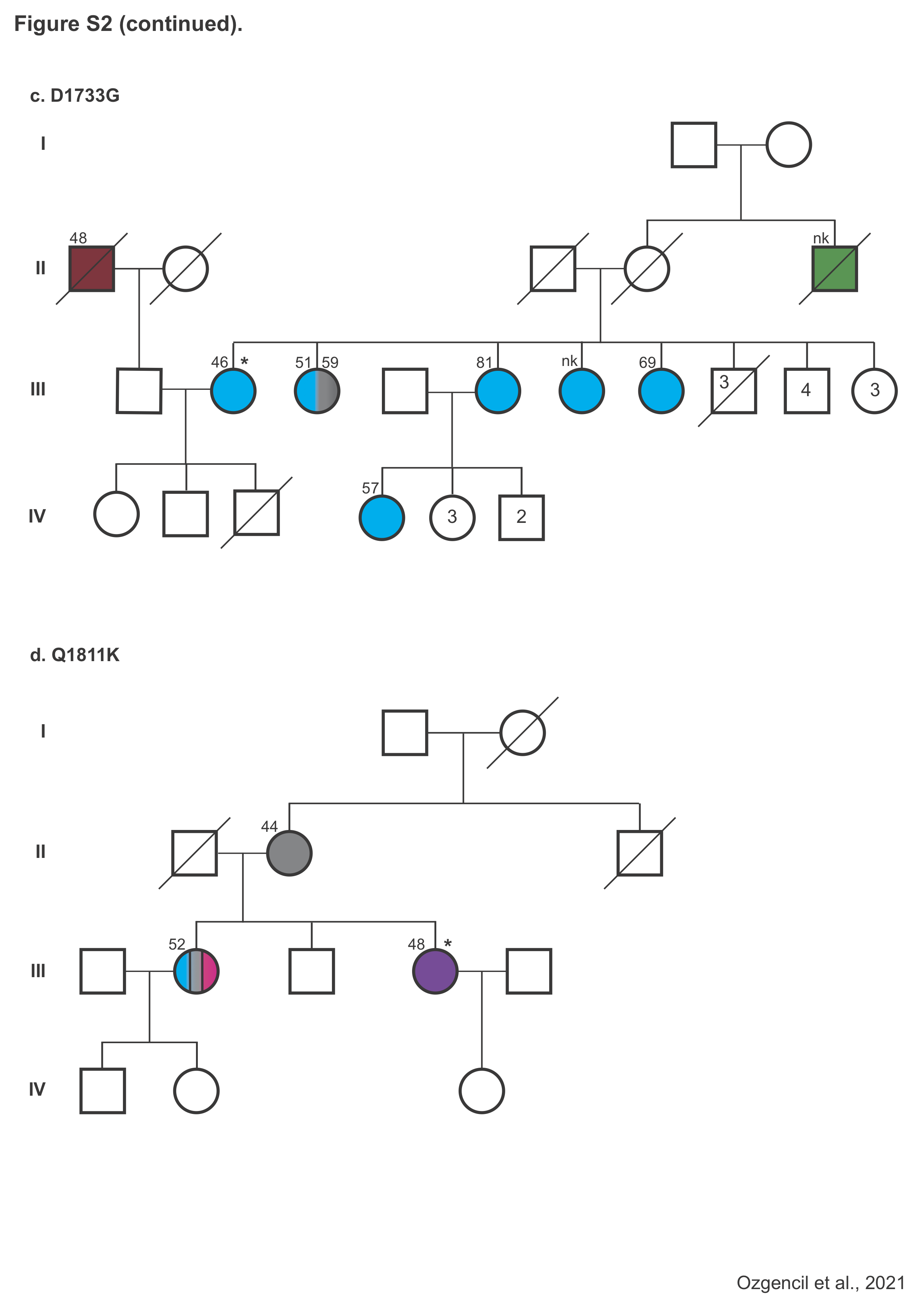

Supplement: S2 Fig — a The six-generation pedigree of the BRCA1 G462R family. b The three-generation pedigree of the BRCA1 Y856H family. c The four-generation pedigree of the BRCA1 D1733G family. d The four-generation pedigree of the BRCA1 Q1811K family. e The five-generation pedigree of the BRCA1 V1687G family. Patient ages at the time of cancer occurrence are located to the upper left of each symbol. Symbols coloured blue or purple indicate patients with breast cancer or ovarian cancer, respectively; pink indicates skin cancer, green indicates lung cancer, orange indicates prostate cancer, dark red indicates leukaemia, and grey denotes cancers with an unknown primary site. A diagonal indicates deceased individuals. Numbers inside symbols indicate multiple individuals. Asterisks (*) identify patients whose biopsies were used for iPS cell derivation. Nk = age at the time of cancer not known. (ZIP) [file pone.0260852.s002.zip › S2cd_Fig.tiff]

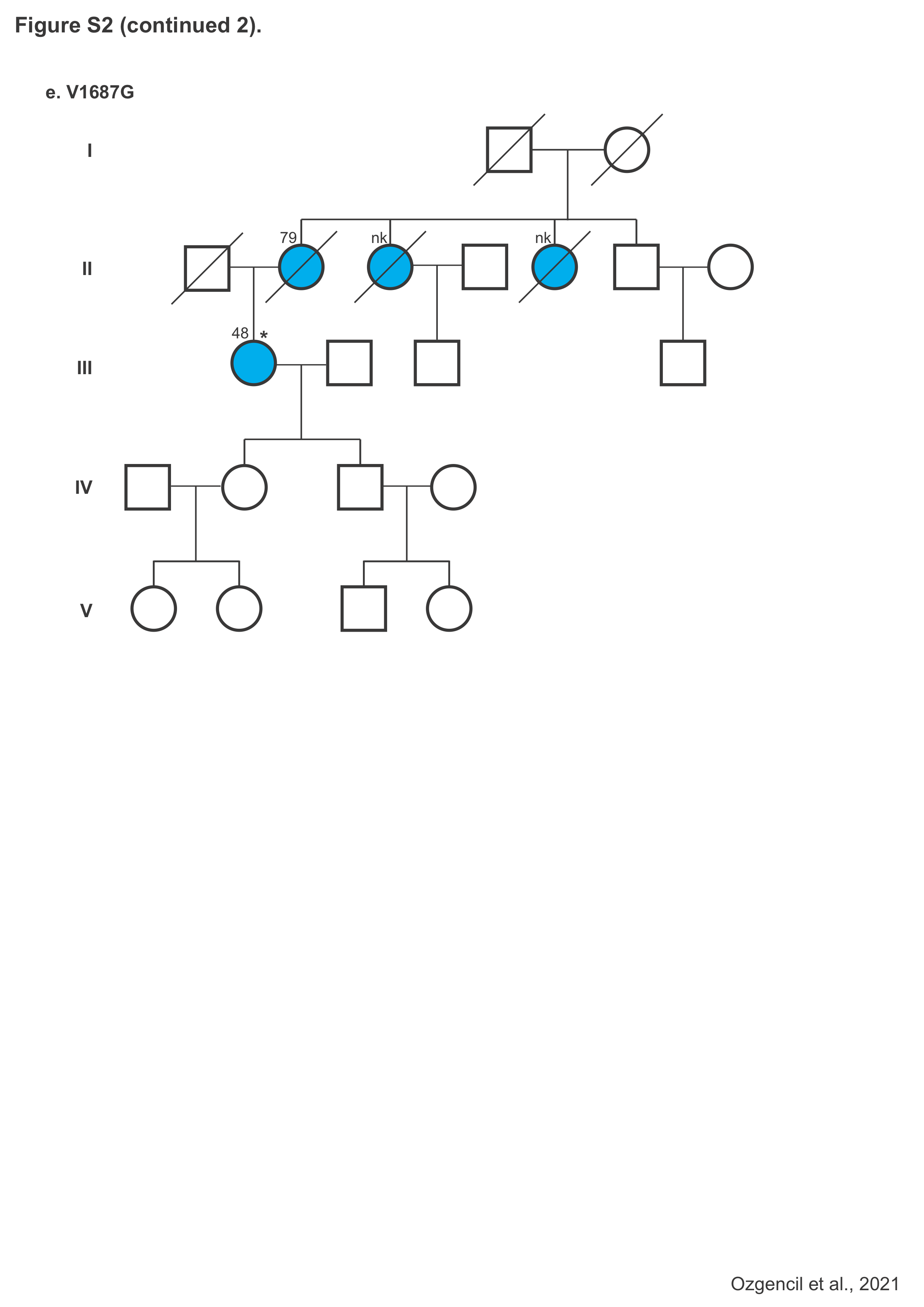

Supplement: S2 Fig — a The six-generation pedigree of the BRCA1 G462R family. b The three-generation pedigree of the BRCA1 Y856H family. c The four-generation pedigree of the BRCA1 D1733G family. d The four-generation pedigree of the BRCA1 Q1811K family. e The five-generation pedigree of the BRCA1 V1687G family. Patient ages at the time of cancer occurrence are located to the upper left of each symbol. Symbols coloured blue or purple indicate patients with breast cancer or ovarian cancer, respectively; pink indicates skin cancer, green indicates lung cancer, orange indicates prostate cancer, dark red indicates leukaemia, and grey denotes cancers with an unknown primary site. A diagonal indicates deceased individuals. Numbers inside symbols indicate multiple individuals. Asterisks (*) identify patients whose biopsies were used for iPS cell derivation. Nk = age at the time of cancer not known. (ZIP) [file pone.0260852.s002.zip › S2e_Fig.tiff]

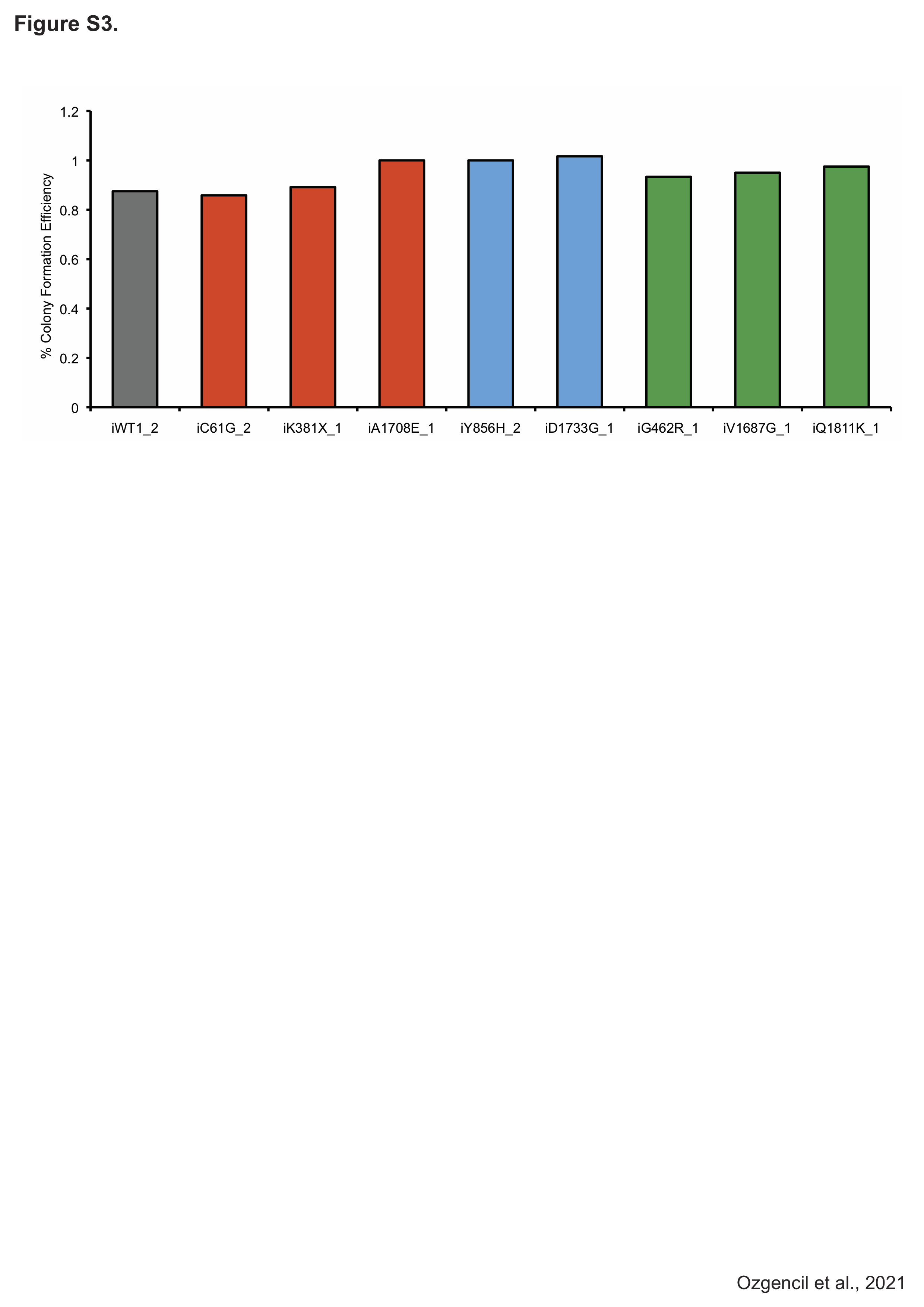

Supplement: S3 Fig — No variation of efficiency to generate induced pluripotent stem cell (iPSC) colonies was observed between all iPS cell lines. (TIFF) [file pone.0260852.s003.tiff]

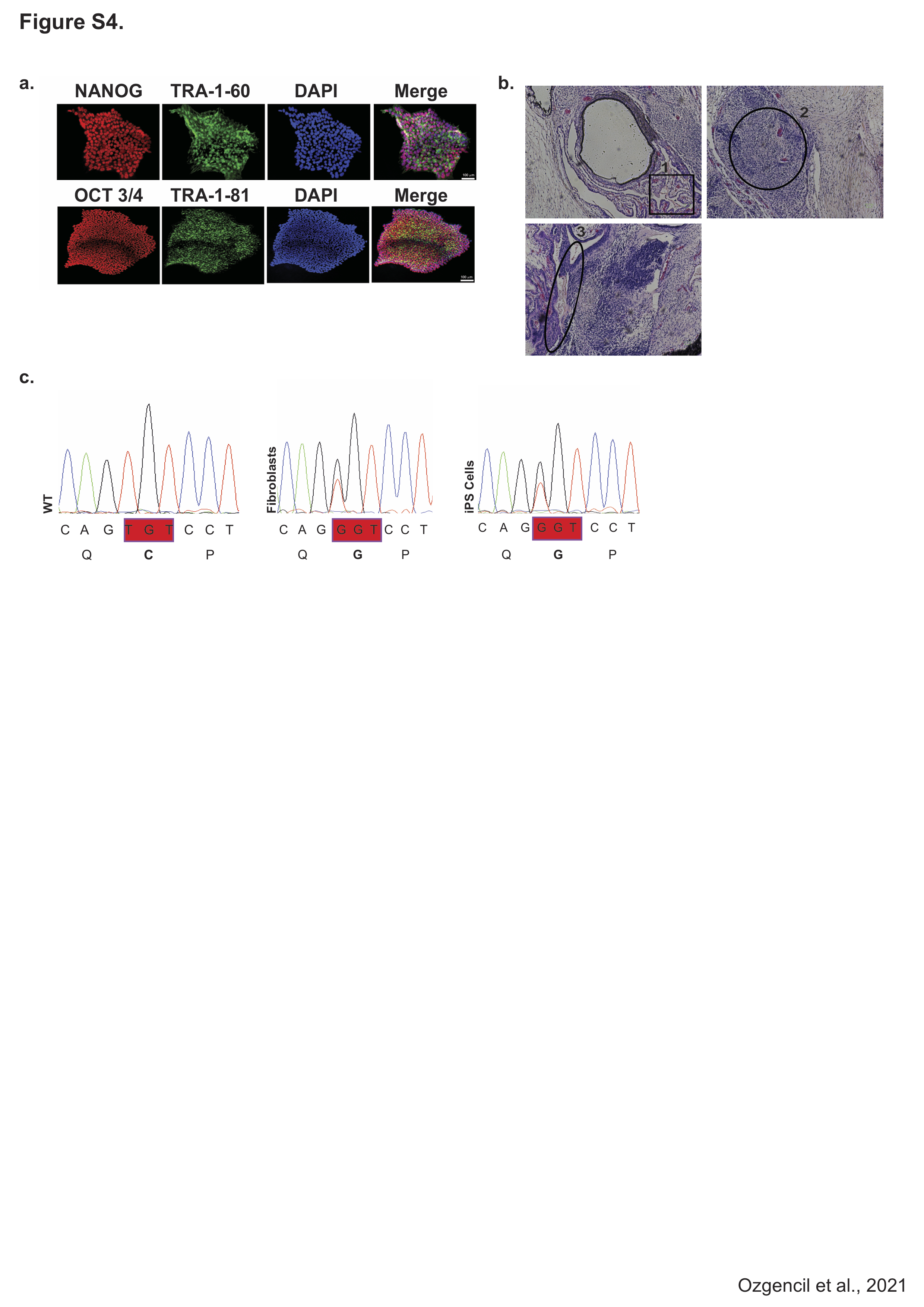

Supplement: S4 Fig — a Representative staining for pluripotency markers for iWT1_2. Nuclei were counterstained with DAPI (blue). Scale bars represent 100 μm. b Representative histological analysis of hematoxylin-eosin-stained images of sections of teratomas derived from iWT1_2 cells, showing all three germ-layers labelled (1 = endoderm, 2 = mesoderm, 3 = ectoderm). c Sanger sequencing showing WT sequence, heterozygous C61G variant present in fibroblasts, and in iPS cells. (TIFF) [file pone.0260852.s004.tiff]

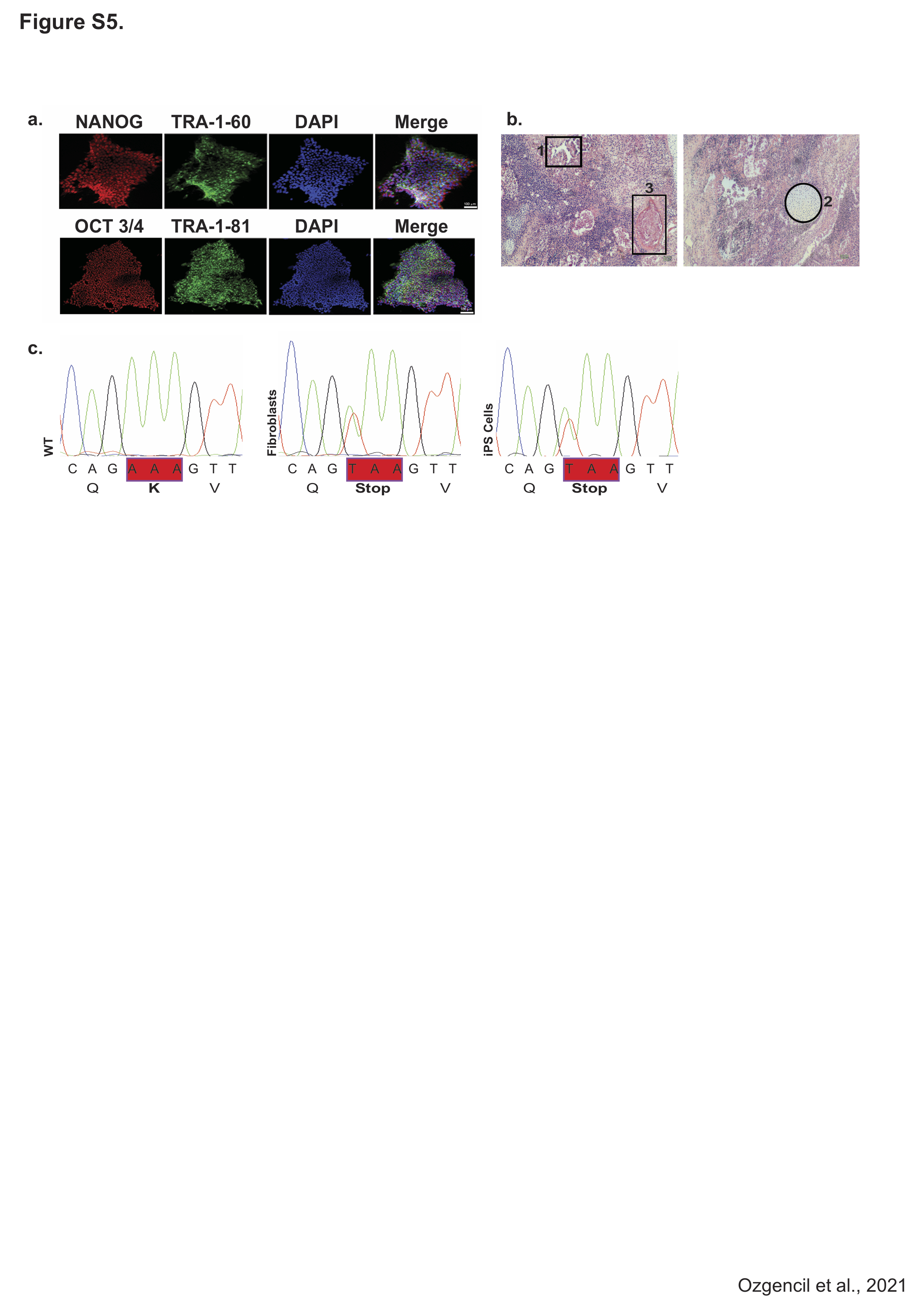

Supplement: S5 Fig — a Representative staining for pluripotency markers. Nuclei were counterstained with DAPI (blue). Scale bars represent 100 μm. b Representative histological analysis of hematoxylin-eosin-stained images of sections of teratomas derived from iPS cells, showing all three germ-layers labelled (1 = endoderm, 2 = mesoderm, 3 = ectoderm). c Sanger sequencing showing WT sequence, heterozygous K381X variant present in fibroblasts, and in iPS cells. (TIFF) [file pone.0260852.s005.tiff]

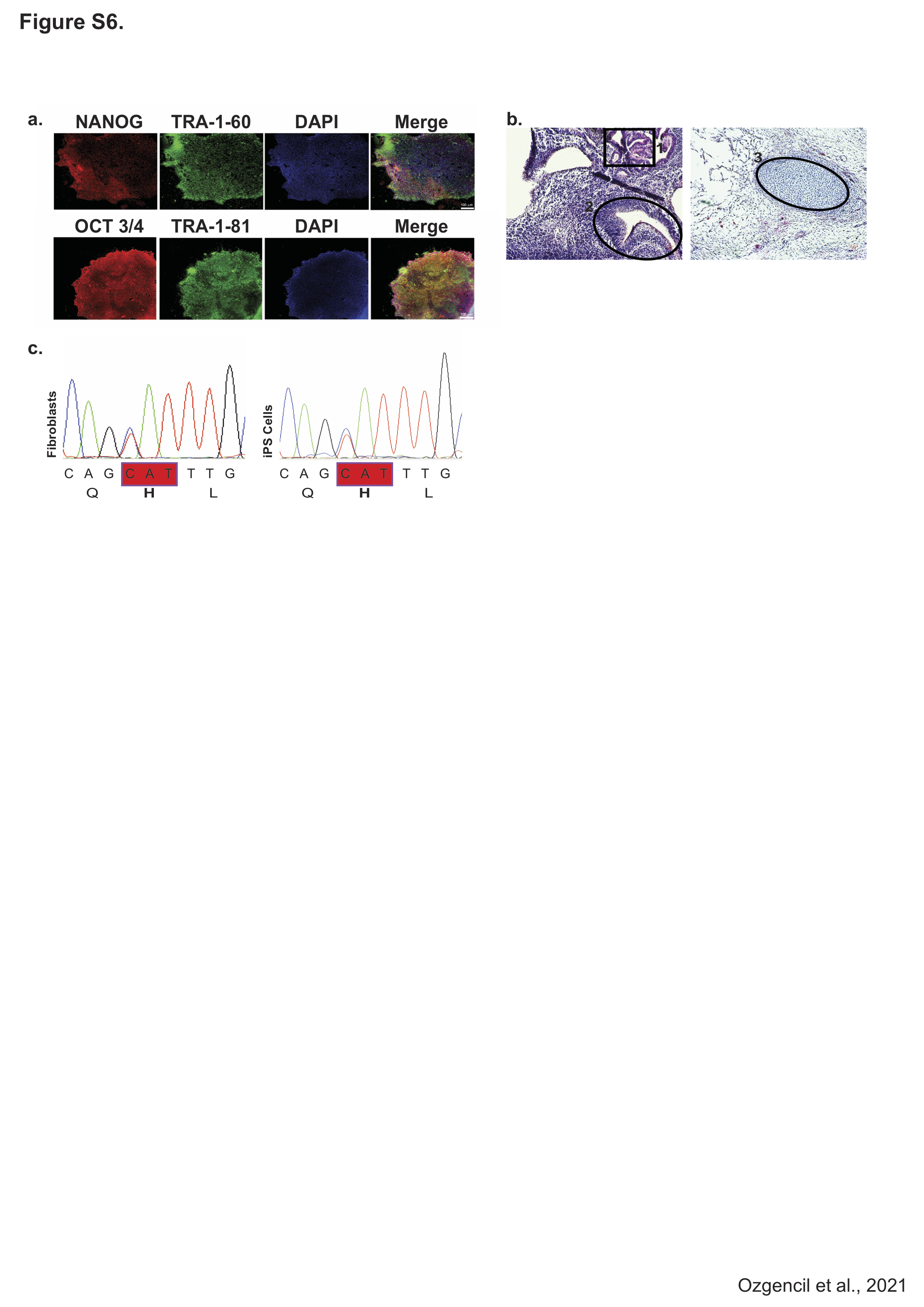

Supplement: S6 Fig — a Representative staining for pluripotency markers. Nuclei were counterstained with DAPI (blue). Scale bars represent 100 μm. b Representative histological analysis of hematoxylin-eosin-stained images of sections of teratomas derived from iPS cells, showing all three germ-layers labelled (1 = endoderm, 2 = mesoderm, 3 = ectoderm). c Sanger sequencing showing WT sequence, heterozygous Y856H variant present in fibroblasts, and in iPS cells. (TIFF) [file pone.0260852.s006.tiff]

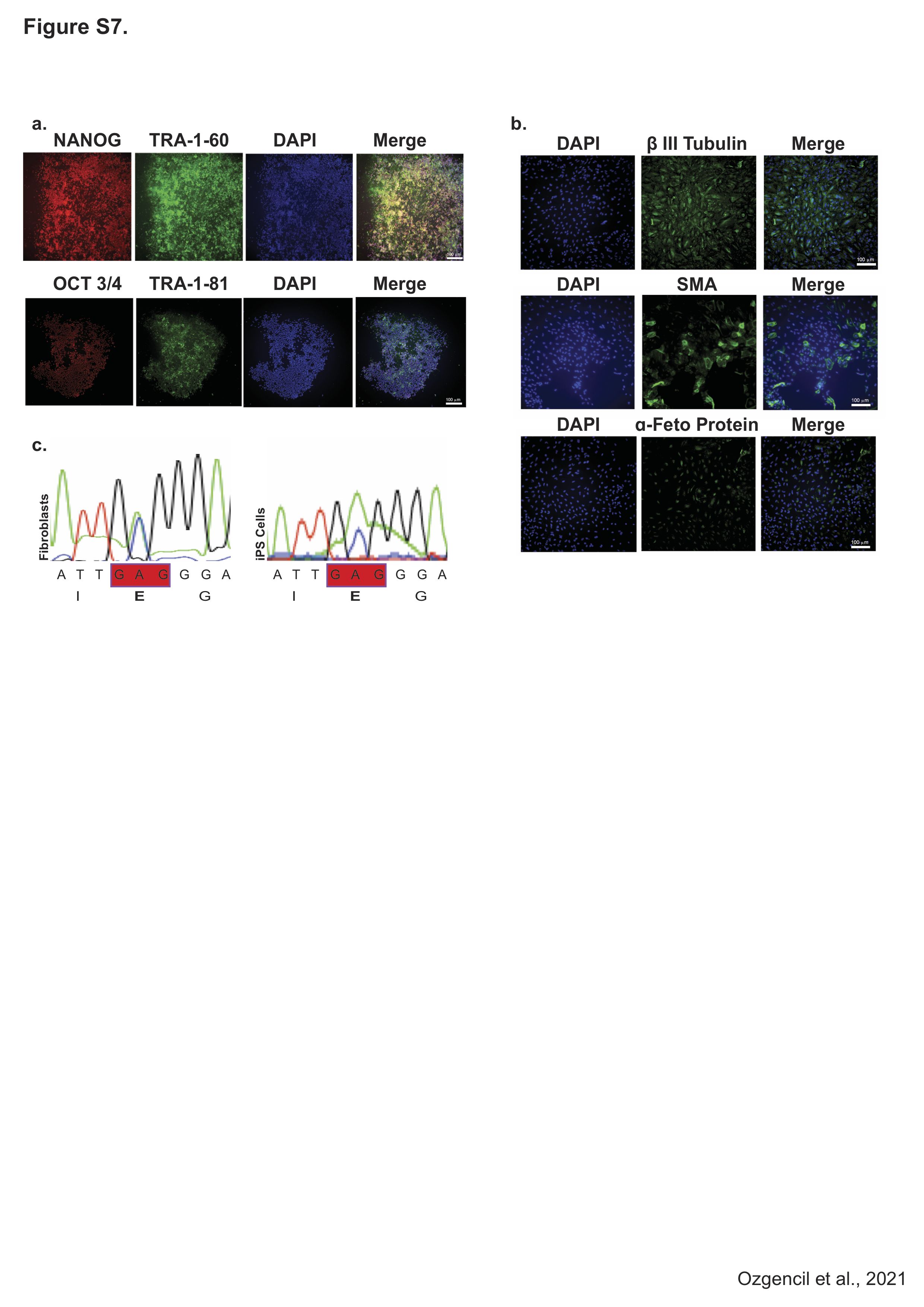

Supplement: S7 Fig — a Representative staining for pluripotency markers. Nuclei were counterstained with DAPI (blue). Scale bars represent 100 μm. b Representative staining of in vitro differentiation potential of iPS cells using specific antibodies against the endodermal marker α-Feto Protein, ectodermal marker β III Tubulin and mesodermal markers α-smooth muscle actin (SMA). Nuclei were counterstained with DAPI (blue). Scale bars represent 100 μm. c Sanger sequencing showing WT sequence, heterozygous A1708E variant present in fibroblasts, and in iPS cells. (TIFF) [file pone.0260852.s007.tiff]

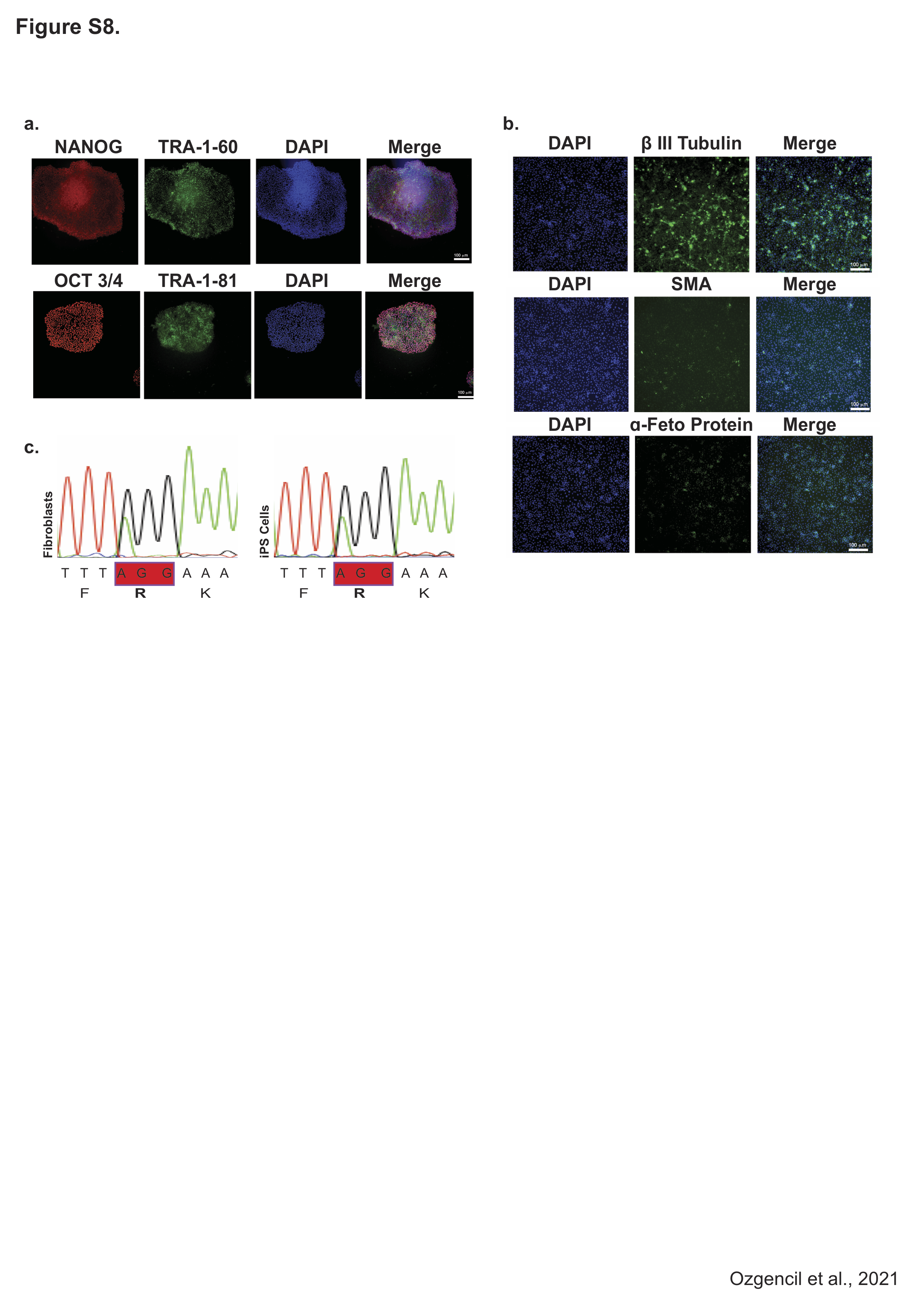

Supplement: S8 Fig — a Representative staining for pluripotency markers. Nuclei were counterstained with DAPI (blue). Scale bars represent 100 μm. b Representative staining of in vitro differentiation potential of iPS cells using specific antibodies against the endodermal marker α-Feto Protein, ectodermal marker β III Tubulin and mesodermal markers α-smooth muscle actin (SMA). Nuclei were counterstained with DAPI (blue). Scale bars represent 100 μm. c Sanger sequencing showing WT sequence, heterozygous G462R variant present in fibroblasts, and in iPS cells. (TIFF) [file pone.0260852.s008.tiff]

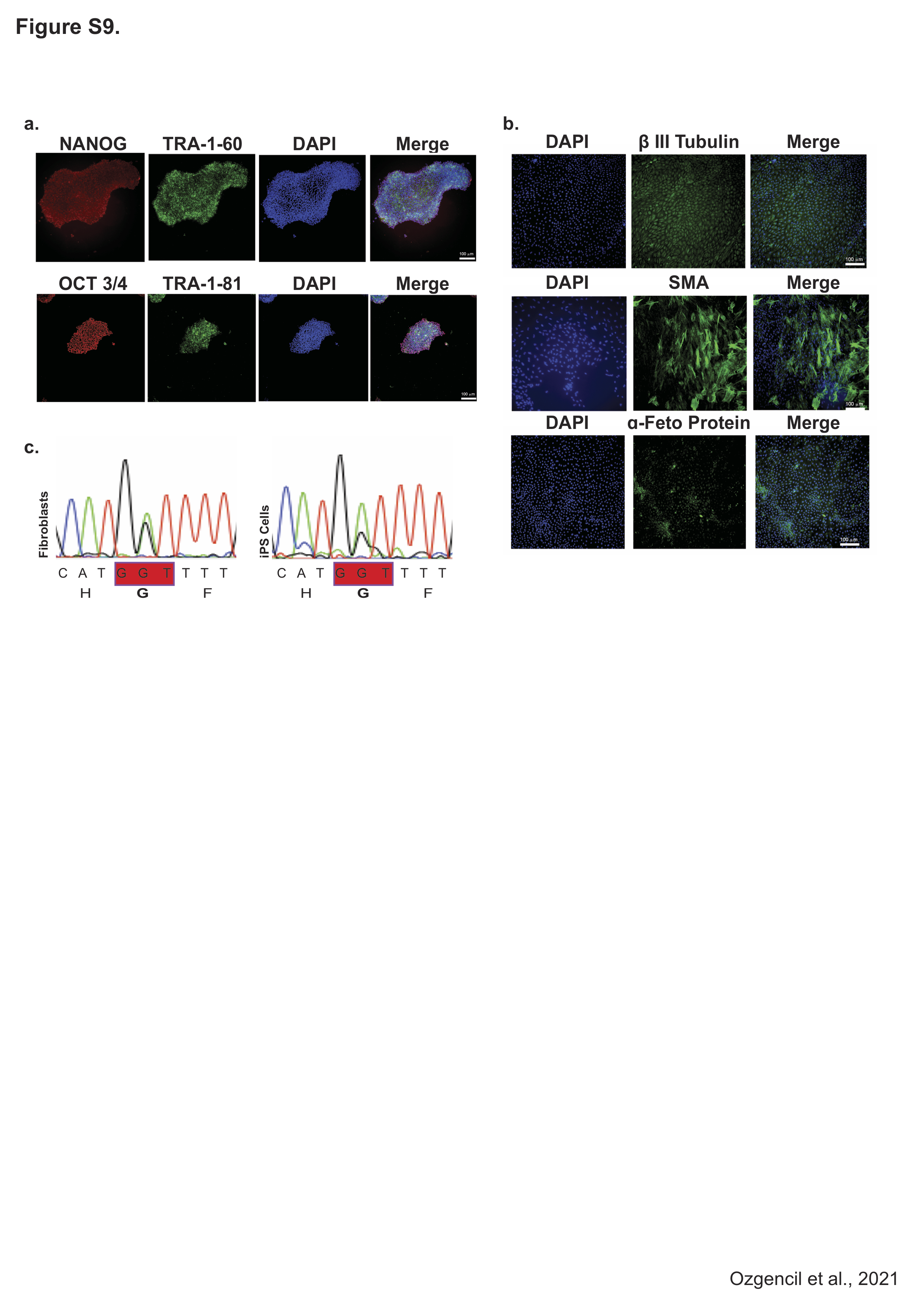

Supplement: S9 Fig — a Representative staining for pluripotency markers. Nuclei were counterstained with DAPI (blue). Scale bars represent 100 μm. b Representative staining of in vitro differentiation potential of iPS cells using specific antibodies against the endodermal marker α-Feto Protein, ectodermal marker β III Tubulin and mesodermal markers α-smooth muscle actin (SMA). Nuclei were counterstained with DAPI (blue). Scale bars represent 100 μm. c Sanger sequencing showing WT sequence, heterozygous V1687G variant present in fibroblasts, and in iPS cells. (TIFF) [file pone.0260852.s009.tiff]

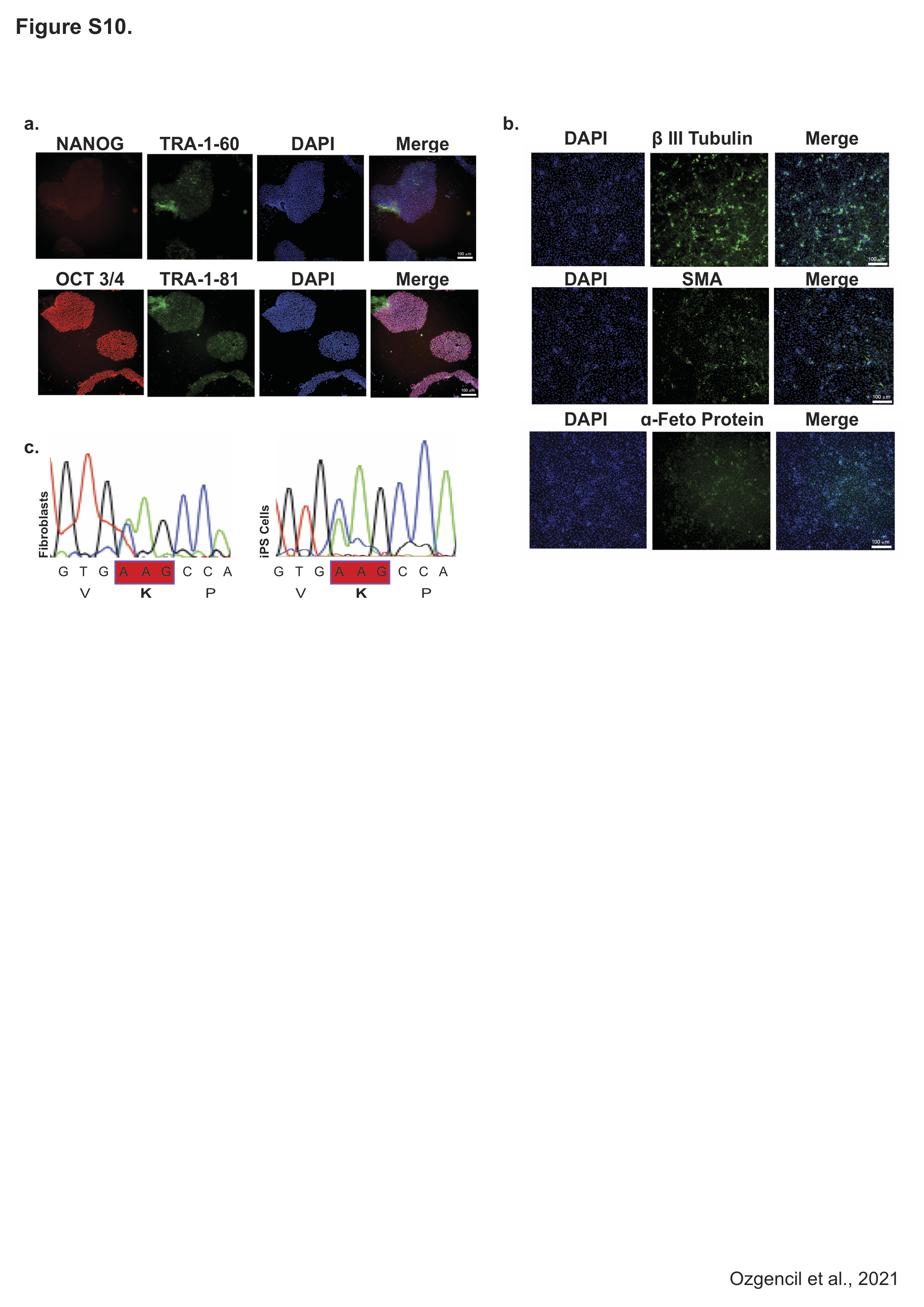

Supplement: S10 Fig — a Representative staining for pluripotency markers. Nuclei were counterstained with DAPI (blue). Scale bars represent 100 μm. b Representative staining of in vitro differentiation potential of iPS cells using specific antibodies against the endodermal marker α-Feto Protein, ectodermal marker β III Tubulin and mesodermal markers α-smooth muscle actin (SMA). Nuclei were counterstained with DAPI (blue). Scale bars represent 100 μm. c Sanger sequencing showing WT sequence, heterozygous Q1811K variant present in fibroblasts, and in iPS cells. (TIFF) [file pone.0260852.s010.tiff]

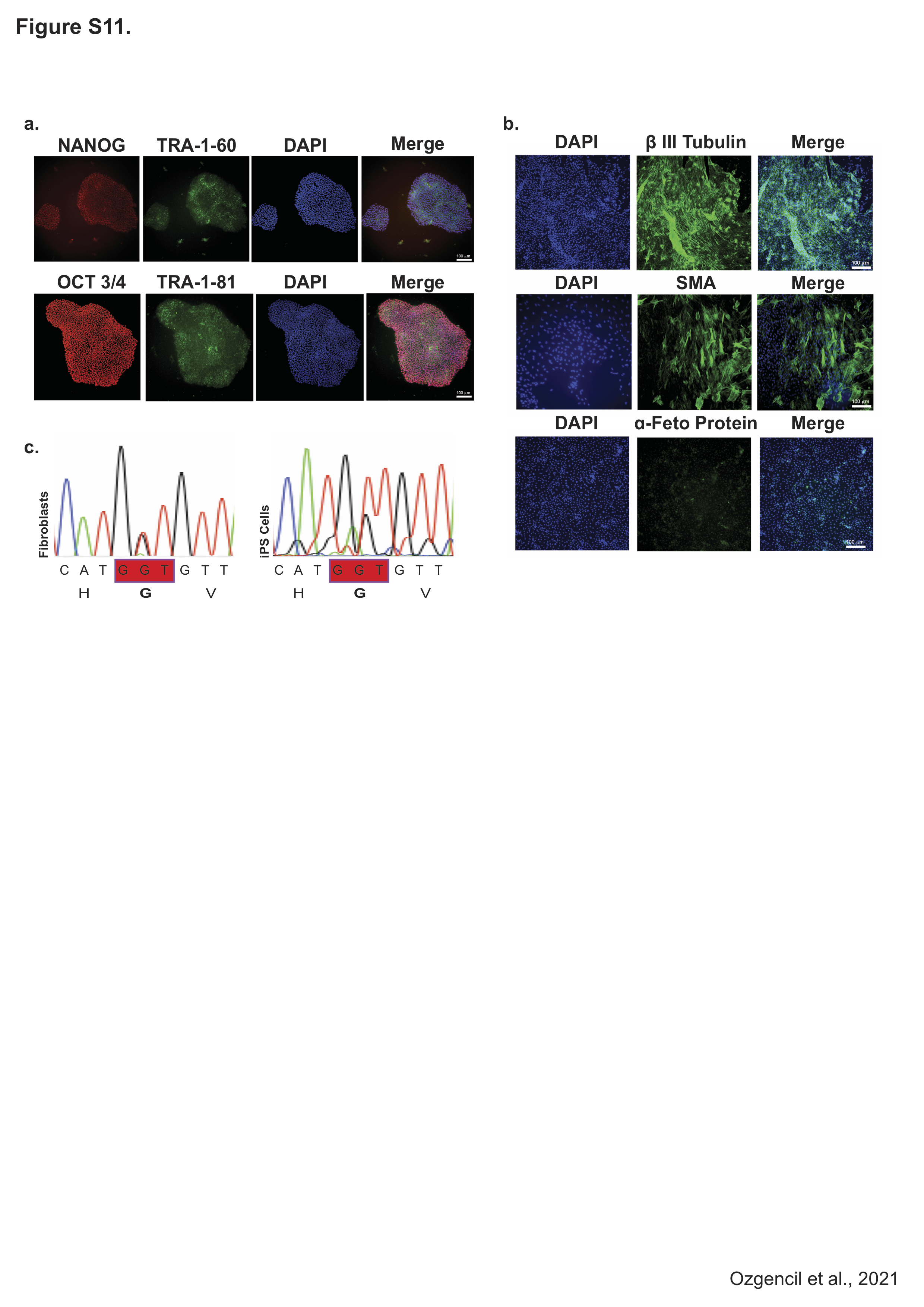

Supplement: S11 Fig — a Representative staining for pluripotency markers. Nuclei were counterstained with DAPI (blue). Scale bars represent 100 μm. b Representative staining of in vitro differentiation potential of iPS cells using specific antibodies against the endodermal marker α-Feto Protein, ectodermal marker β III Tubulin and mesodermal markers α-smooth muscle actin (SMA). Nuclei were counterstained with DAPI (blue). Scale bars represent 100 μm. c Sanger sequencing showing WT sequence, heterozygous D1733G variant present in fibroblasts, and in iPS cells. (TIFF) [file pone.0260852.s011.tiff]

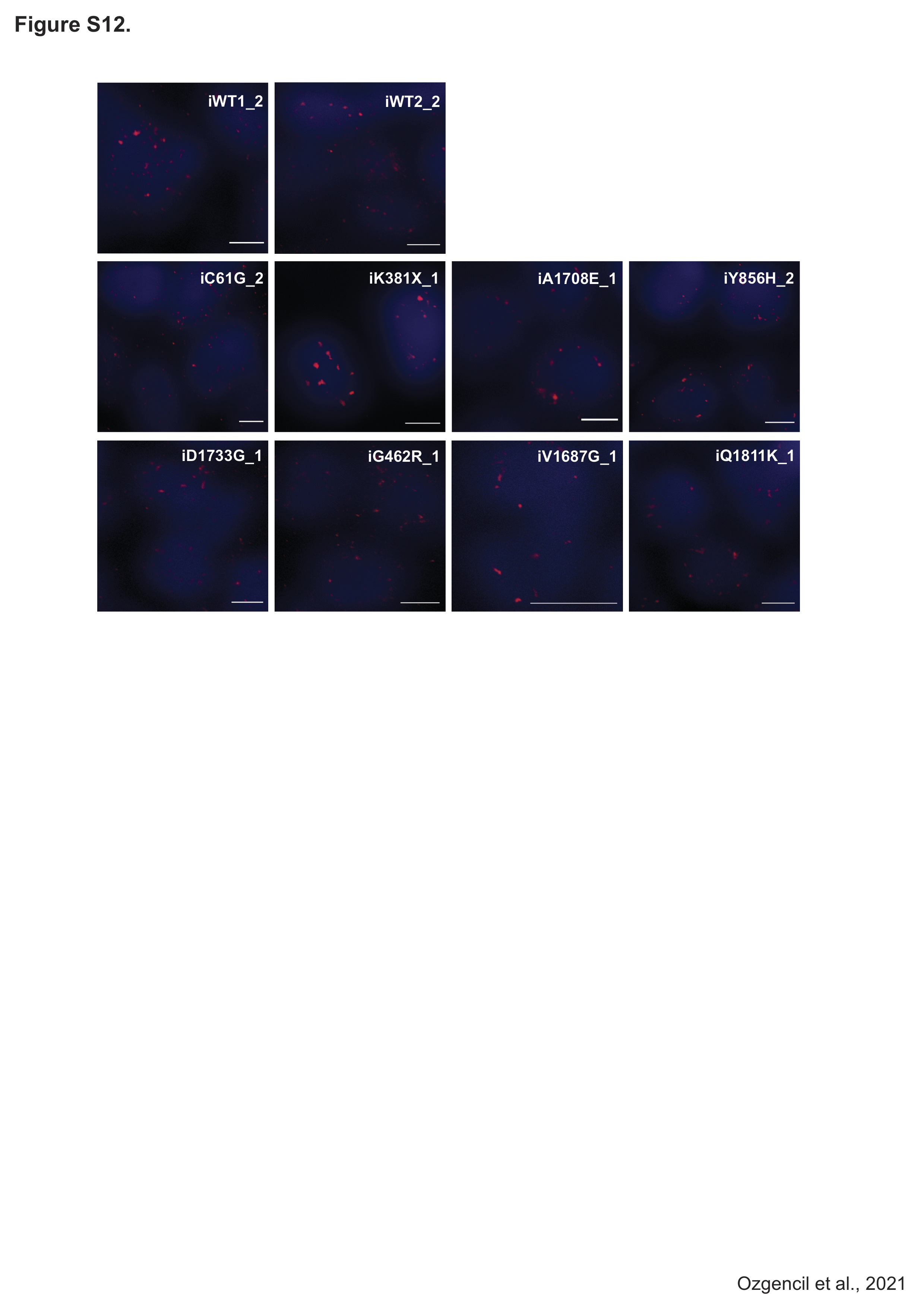

Supplement: S12 Fig — Immunofluorescence staining for γH2AX nuclear foci formation following ionising radiation exposure. Nuclei were counterstained with DAPI (blue). Scale bars represent 10 μm. (TIFF) [file pone.0260852.s012.tiff]

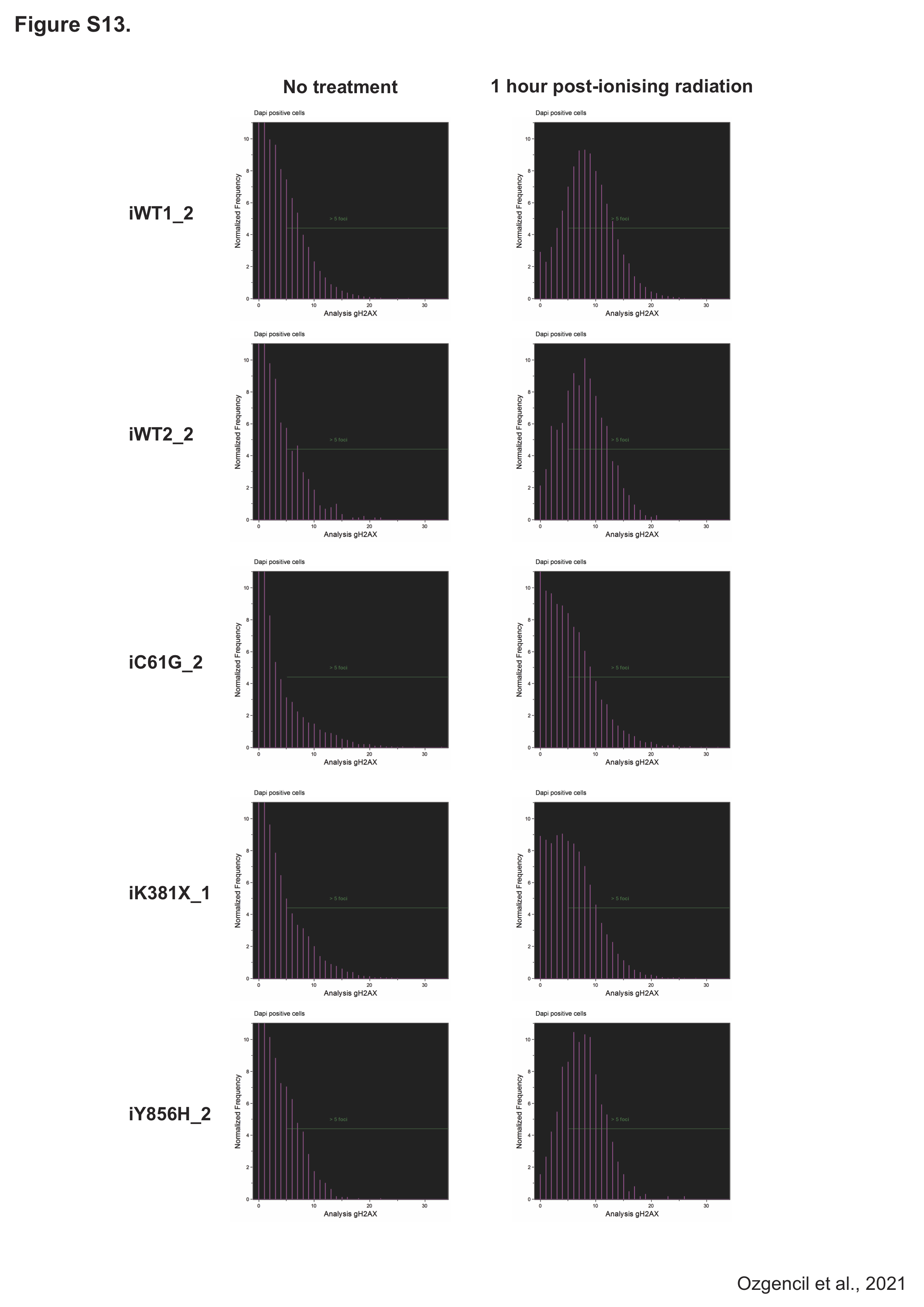

Supplement: S13 Fig — Representative foci quantification graphs for iWT1_2, iWT2_2, iC61G_2, iK381X_1 and iY856H_2 iPS cells before and after IR treatment. (TIFF) [file pone.0260852.s013.tiff]

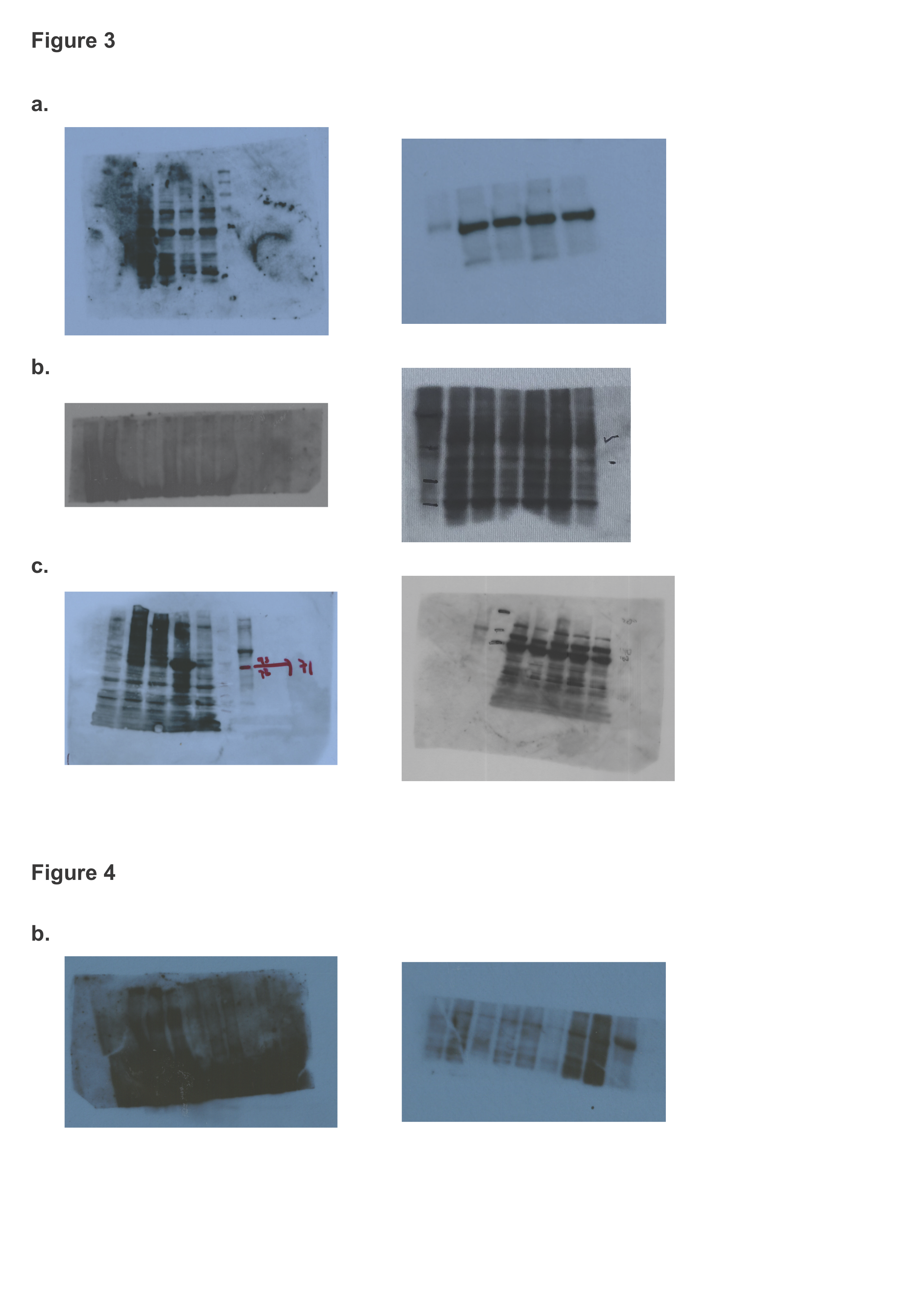

Supplement: S1 Raw images — (TIFF) [file pone.0260852.s016.tiff]
